# Future Intent to Run and Running Performance of Students Exposed to a Traditional versus an Autonomy Supportive Motivational Running Program

**DOI:** 10.1155/2013/471657

**Published:** 2013-03-25

**Authors:** Andrea Silva, James C. Hannon, Barry Shultz, Leslie Podlog

**Affiliations:** Department of Exercise & Sport Science, University of UT, Salt Lake City, UT 84112, USA

## Abstract

*Background*. The study's primary purpose was to investigate whether an autonomy supportive motivational climate in a running program would increase future running intent among high school students. A secondary purpose was to examine whether the program would increase individual performance in the Cooper 12-minute run. *Methods*. Students participated in a 4-month running intervention program which included four timed runs, one per month, and a future intent questionnaire prior to the start of the timed runs and following the last run. *Results*. Factorial repeated measures ANOVA revealed significance regarding future intent (*P* = .026) at both schools. Factorial repeated measures ANOVA indicated differences between the runs at both schools (*P* < .001). Paired samples *t*-tests were conducted to look at significance with paired runs. Results revealed significance in two of the six pairs at the treatment school, notably between the first and last timed runs (*P* = .004). Only one pair was found to be significant (*P* < .001) with the control school. *Conclusion*. At both schools, the overall number of laps increased as well as future intent to run scores. The results do not support evidence of a greater effect from the autonomy supportive environment over a traditional environment.

## 1. Introduction

Since the 1970s the prevalence of obesity among adolescents in the US has doubled and it has more than tripled among children aged 6–11 [1]. In fact, one out of every six individuals aged 2–19 are obese in the US [2]. Obesity in childhood and adolescence increases risks of developing cardiovascular disease, orthopedic issues, and psychosocial problems [3]. In an effort to reduce the prevalence of obesity and associated health problems among youth, many schools have begun implementing running programs [4].

 Running has been recommended by governmental agencies, including the National Institutes of Health and the Centers for Disease Control and Prevention, as well as professional organizations such as the American Alliance for Health Physical Education Recreation and Dance as an integral element in physical education (PE) curricula across all levels of schooling [5]. Running has many health enhancing benefits including the prevention and management of diabetes and heart attacks, lowering blood pressure, and enhancing weight loss. Running also improves bone health and coordination, while boosting the immune system and improving mood [6–8].

 Several studies focusing on the outcomes of compulsory running programs at elementary and middle schools have been published [9–14]. However, there has been little research conducted on high-school-aged students and running programs [15]. This is unfortunate because many health-related behaviors cultivated in late adolescence track into adulthood [16].

 The research conducted at elementary and middle school levels has concluded that intrinsic motivation (i.e., fun and pleasure derived from the activity itself) and enjoyment is a key to program success [14]. Running is traditionally teacher-led and usually perceived as negative or not enjoyable by students [10, 17]. Hopple and Graham [9] found that students created a myriad of excuses to “dodge the mile” because they disliked running so much. Xiang et al. [4] found that students who did not enjoy a running program disliked it because of boredom, discomfort and running too much or too often. The students who enjoyed a running program did so because they perceived it as being fun; they enjoyed setting goals and achieving those goals. However, Xiang et al. [4] found in their research that as run times improved, participants became less motivated to run over the school year. Xiang et al. [14] found that a participant's future intent for running was significantly related to his or her 1-mile run performance. However, Xiang et al. [14] felt that it was not the physical act of running the participants disliked, but the delivery system of the running program itself that was affecting the participants' motivation. Students were not allowed to talk to peers/friends, listen to music, or run in different locales. Xiang et al. [14] suggested that in the future, students should be allowed to talk with their peers/friends, music should be implemented, and locations and workouts for the students should be varied. The research to date has not directly compared outcomes of different types of running programs. This step is important in order to determine if different approaches, such as using various motivational techniques, will result in better outcomes versus an approach such as a traditional teacher-led running program in which students experience little autonomy or variety in their running involvement. 

 Given recent research and meta-analytic findings highlighting the importance of an autonomy supportive motivational climate in health promotion [18] and PE settings [19], a self-determination theory (SDT) perspective was used to guide the current study. A central tenant of SDT is that the nature of the social context will influence an individual's motivation, well-being, and task performance. According to SDT researchers, [20, 21] autonomy supportive environments (i.e., social contexts supportive of choice, initiation, and understanding) as opposed to controlling environments (i.e., social contexts that are rigid, pressuring, and dictating) will facilitate intrinsic motivation, positive psychological well-being (e.g., self-esteem), and enhanced behavioral outcomes (e.g., persistence and performance). Consistent empirical support for these contentions has been found in various studies [18–21]. For instance, Cheon et al. [19] found prospective evidence that Korean students who received autonomy supportive instruction during PE lessons self-reported a host of positive outcomes such as greater classroom engagement, skill development, academic achievement, and future intention for physical activity in comparison to control students receiving regular instruction.

Given the prevalence of running programs in adolescent PE and the dearth of research among high-school-aged cohorts focused specifically on running programs [15], it is apparent that further research is needed. In addition, while past research has focused on the influence of autonomy supportive environments on perceptions of motivation and intent for future exercise, research is needed focusing on objective measures of exercise performance (e.g., Cooper 12-minute run test). The primary aim of this study was, therefore, to determine if an autonomy supportive motivational climate for a running program for high school PE classes would result in participants having a greater intention to run in the future as compared to students in a controlled, traditional teacher-led program. It was hypothesized that students who were exposed to the autonomy supportive motivational climate (treatment setting) would demonstrate a higher future intent to run compared to students in a traditional teacher-led running program in which no choice regarding one's running involvement was offered (control setting). The secondary aim was to determine if an autonomy-supportive motivational climate for a running program would result in enhanced individual performances in the Cooper 12-minute run test versus students in a controlled, traditional teacher-led running program. It was hypothesized that students who were exposed to the autonomy supportive motivational climate would demonstrate greater individual performances in the Cooper 12-minute run test. 

## 2. Methods

### 2.1. Participants

The participant recruitment pool consisted of 500 students aged 14–19 years old enrolled in ten Fit-for-Life classes at two high schools located in the Southwestern United States. Five classes in each school participated with class enrollments ranging between 45 and 55 students. Participation was voluntary, and a total of 247 (92 intervention school; 155 control school) students chose to participate in the study. Both schools were located in the same school district and possessed similar demographics, facilities, and enrollments of approximately 1500 students in the 10th–12th grades. This study was approved by the University Institution Review Board for studies involving human participants and informed assent, and parental permission was obtained.

### 2.2. Instruments

#### 2.2.1. Running Programs

The actual running regimen was the same for both schools and included daily workouts for each teacher with detailed instructions regarding the length of time and locations for each run or activity. Each class was approximately 90 minutes in length and met approximately five times every 2 weeks. Running duration increased every 2 weeks over the course of 14 weeks, progressing from 6 minutes. to 35 minutes, for both research groups throughout the running program. In addition to increasing the amount of time the participants ran, fartlek and interval training were used as part of the running program. The control group experienced a traditional teacher-led program using a direct instructional style of teaching in which students were merely required to run for the specified amount of time by the teacher. In the control condition, students experienced little autonomy as there was no choice regarding the duration of running or the route one could take. In contrast, the intervention group experienced an autonomy supportive motivational climate in which participants had the opportunity to keep a running logbook, set self-selected goals (with appropriate instruction), listen to music of their choice while running, and choose a partner/group to run with at a self-selected pace. These autonomy supportive techniques have been shown to improve intrinsic motivation, enjoyment, and intention in participants [13, 21–27]. In regard to goal setting, the participants were taught a lesson on types of goals and how to set goals. The participants set personal goals for the overall running program, weekly goals and daily goals. These goals were checked in class and reevaluated throughout the running program. Participants used their log-books to help track progress towards their goals. Lessons were taught and readings were provided on the benefits of running, proper stretching, basic nutrition and hydration for running, and information on running clothing and finding the right running shoe. Participants also received a schedule of local road races in the county. The aim was to make running more meaningful to the participants by increasing their perceptions of an internal locus of causality (i.e., a greater sense of personal control) and subsequent future intent to run.

#### 2.2.2. Future Intent

This construct was assessed using three questions which were adapted from questions used in similar studies [4, 13, 28]. The questions asked in this study were the following: (a) “when you are not enrolled in PE next semester, will you continue to run on your own?,” (b) “do you plan to run outside of PE?” and (c) “Do you have plans to continue running in the future”? All three questions were answered using a Likert scale with answers ranging from 1 = *not at all* to 5 = *very much*. The responses were averaged to form a single intent score with higher scores indicating a high level of intention and lower scores indicating a low level of intention.

#### 2.2.3. Running Performance

Running performance was assessed using the Cooper 12-minute walk/run test. The Cooper 12-minute walk/run test has a reported correlation of. 90 between VO2 max and the distance covered in a 12-minute walk/run [29]. The objective of the test is to run as far as possible around a track in 12 minutes. The 12-minute walk/run was used in lieu of the more conventional mile-run test because all students run at the same time for the full 12 minutes; therefore, the slowest runners in the class are not left to be watched by the fastest runners who would have already competed the test. 

### 2.3. Procedures

**Figure 1:**
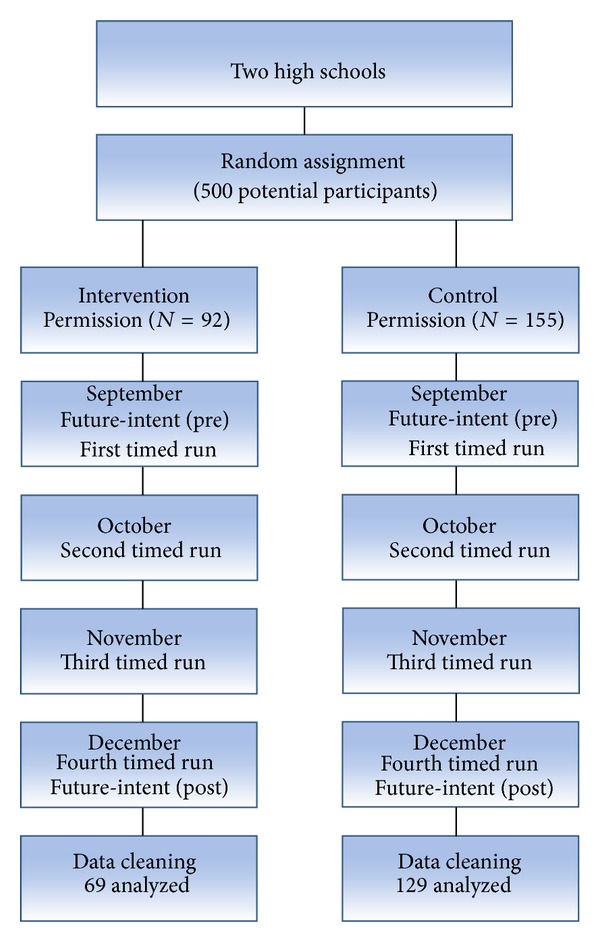
Data collection flow chart.

Informational and organizational meetings were held to discuss procedures, program curriculum, and purposes for the study. In attendance were the Fit-for-Life teachers from both high schools, the PE and Wellness Director of the school district, and the researchers. One school was randomly chosen as the control (traditional) school and the other school was designated as the treatment (motivational) school. Teachers received the complete running intervention curriculum as well as researcher contact information. Class curriculum was not altered with the implementation of the running programs other than an increasing specified time segment that was set aside during each class period for the participants to run. Classes were visited periodically (approximately eight times for each school) during the program to make sure that lessons and protocol were being followed. Prior to the start of data collection, parental permission and child assent were obtained. Each participant completed the Cooper 12-minute run four times during the fall semester. All timed runs took place on the high school's indoor tracks to control environmental conditions. Both of the tracks were identical in size, requiring 10.5 laps to equal one mile. The first run was conducted in the beginning of the school year at the start of the investigation, occurring during the second week of September. Successive timed runs were conducted one time each month, with the final run in December. All participants started at the designated start line on their respective indoor track for each run. The participants ran around the track for 12 minutes until the PE instructor blew a whistle indicating to the participants to stop running. Participants reported to their teacher how many times they ran around the track plus any extra distance they covered based on where they started (one-quarter, one-half, or three-quarters of the way around the track) when the whistle blew. The teachers, with help of research assistants, counted and recorded each student's laps, thus reducing the potential of reporting bias by the individual students to only the extra distance covered beyond a full lap. Participants completed the future-intent questionnaire prior to the first timed run in September and after the final run in December.  Figure 1 depicts a flow chart of the studies procedures.

### 2.4. Data Analysis

Participants must have completed the future-intent questionnaire and have run three of the four Cooper 12-minute run tests including the first and last timed runs. If a participant did not meet this requirement, they were deleted from the final analysis. Statistical analysis for all data in this study was conducted using SPSS version 15.0 (SPSS, Chicago, IL).

Two separate one-way ANOVAs were used to compare baseline data of students who completed the study requirements and those who did not. In order to input missing run values, two separate regression analyses were conducted to predict missing October and November run values. Out of the total sample, 2 (*n* = 1 for treatment, *n* = 1 for control) runs were missing from October and a total of 11 (*n* = 3 for treatment, *n* = 8 for control) runs were missing from November.

A 2 (treatment school, control school) × 2 (males, females) × 2 (pretest, posttest) factorial mixed repeated measures analysis of variance (ANOVA) was conducted to determine if the motivational running intervention program for high school Fit-for-Life students would increase future intent to run compared to students in a traditional teacher-led program. 

To determine the effect of the motivational intervention on the Cooper 12-minute run performance, a 2 (treatment school, control school) × 2 (males, females) × 4 (monthly timed runs) factorial mixed repeated measures ANOVA was conducted to determine if the motivational running intervention program would result in enhanced individual performances compared to students in a controlled traditional teacher-led running program. Significance was set at *α* = 0.05 for all tests.

## 3. Results

After screening for missing data, the final sample consisted of 198 participants (*n* = 69 treatment school; *n* = 129 control). The majority of the participants were 15 years old (85.4%), enrolled in 10th grade (88.4%), and of Caucasian descent (88.9%). There was no statistical significance found in the baseline future intent to run and Cooper 12-minute run test scores between students who completed the study requirements and those who did not.

### 3.1. Future Intent to Run

Descriptive data for pre- and posttest scores are displayed in Table 1. Results revealed that over time, students' future intent to run increased significantly, regardless of the type of motivational climate *F*(1,197) = 5.07, *P* = .026 (Table 1). However, there was no statistical significance found when looking at the interaction of time and motivational climate, time and gender, nor time, motivational climate, and gender.

### 3.2. Cooper 12-Minute Run Test

Results revealed that over time students' laps ran increased significantly, regardless of the type of motivational climate *F* (3, 193) = 7.96, *P* < .001. The time by climate interaction was also significant *F* (3, 193) = 8.58, *P* < .001 starting with *M* = 11.49 and *M* = 14.01 for the treatment and control schools, respectively, in September, which increased to *M* = 13.43 and *M* = 14.39 respectively, in December. The results of the between subjects effect revealed that there were a significant *F* (1, 193) = 36.76, *P* < .001, difference between the genders regardless of schools (Table 2) as well as a difference between schools *F* (1, 193) = 19.97, *P* < .001. The interaction effect of time, motivational climate, and gender was not statistically significant.

A paired-samples *t*-test was conducted to evaluate simple main effects of the program. The difference in pattern gives a sense of the differences across time at each of the schools. In order to account for increased alpha error rate, an alpha-adjustment was conducted with significance at alpha = .05/12 = .004. The results indicated that the mean lap scores (*M* = −.699, SD = 1.86) for only the first paired run (September and October) at the control school (*M* = .556, SD = 2.35) were significant; *P* < .001 (Table 3). At the treatment school, the October–December and September–December were both significant (Table 3). Most notable is the third pair (September and December), which looked at the mean difference between the first and last runs. At the treatment school (*M* = −1.887, SD = 5.25), there was a significant difference (*P* = .004) between the September and December runs. This is important to note because there was no significance found for this same particular pair in the control group. This result assists in answering the research question of whether or not the motivational climate assisted with increased run performance with the Cooper 12-minute run test. Based on the current research results, there was a significant difference found between the first run in September and the final run in December only for the treatment group.

**Figure 2:**
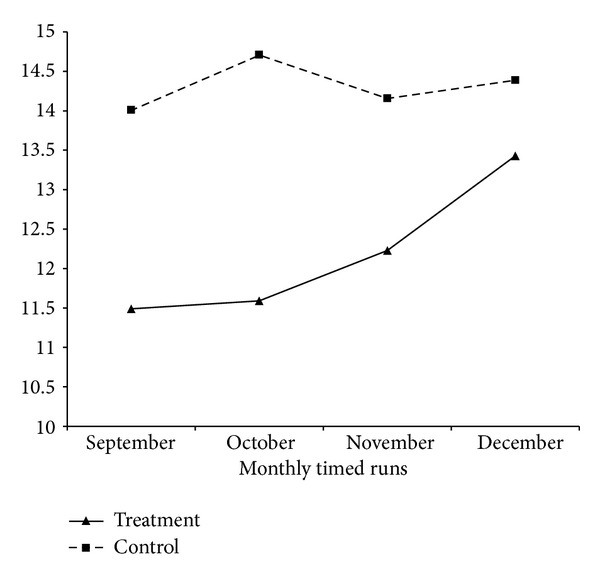
Time × climate interaction.

Furthermore, when investigating the time by climate interaction, data revealed an increase in the average number of laps run every month over time in treatment participants from the first time run (September) to the last timed run (December; see Figure 2). However, a similar progression of average laps run was not seen in the control group from September to December. Data revealed an increase from the September to October runs; however, there was a decline in laps run from October to November. Finally, the average number of laps run did increase again from November to December.

## 4. Discussion

The purpose of this study was to determine if an autonomy supportive motivational climate for a running program would result in participants having a greater intention to run in the future and faster times on the Cooper 12-minute run test than students in a traditional controlled teacher-led program. It appears that the 4-month running intervention program had positive results at both schools.

With regard to future intent, the motivational climate did not differentially affect participant's intention to run in the future. Overall at both schools, future intent to run increased, regardless of the climate they experienced, from the beginning of the running intervention program to the conclusion of the program, and test results revealed that overall future intent to run was found to be significant. These results do not support our hypothesis that the participants exposed to the autonomy supportive motivational climate would report higher future intent scores than those in the control group. Although the treatment group reported a higher future intent difference (from pre- to posttest results) than the control school, both schools reported higher future intent means in the posttest compared to the pretest. These findings appear inconsistent with previous SDT research highlighting the benefits of an autonomy supportive environment in increasing future intent to exercise in PE settings [19, 22]. One possible explanation is that the motivational strategies used to foster perceptions of autonomy were not consistently reinforced throughout the duration of the running program. Hence, the treatment may have engendered insufficient perceptions of autonomous running involvement. Another plausible reason for the increased intent to run at both schools could be due to the competitive nature of the adolescent cohort examined in this study. Even though much emphasis was placed on not making the environment competitive at either school given the age of the participants perhaps they were competitive with their peers as well as with their own previous laps run and wanted to improve from their previous timed run. Although there was no formal goal setting at the control school, it seems possible that students in the teacher-controlled environment also set personal goals. Such goal setting may have increased intentions to run in the future at both schools.

Findings from this study also contradict those by Xiang et al. [4]. Using a sample of elementary aged participants, Xiang et al. [4] found that the older the student was, the lower their future intent to participate in PE became. The highest future intent scores were reported by the lower grade levels and the lower future intent scores were reported by 5th graders. This finding does not support the current findings. Given Xiang et al.'s [4] findings, one would expect to see lower future intent scores across the board given the fact that the participants in this study were high school students. However, the findings regarding future intent to run in this study are positive in so far as adolescents in both schools reported greater intent to run from pre- to posttests. Therefore, the autonomy supportive motivational climate did not have much of an impact on future intent to run as originally hypothesized as both groups overall increased their future intent to run. Although previous research has been conducted investigating future intent to run, all the research that has been found has been conducted on elementary school students. Therefore, the possible reasons for the current research findings are only possible explanations and not based on previous scientific findings. At the treatment school, perhaps the tactics that were implemented (i.e., log-books, lessons and informational handouts) were not specifically geared toward (not of interest to) the high-school population and therefore did not impact overall future intent for those participants. Evidently, the running program itself had more of an impact on both schools' participants' future intent to run than the motivational strategies, which were only implemented at the treatment school during the program, given that overall future intent means increased from the pre- to posttest at both schools. Given that the biggest gain in running performance occurred during the last month, it is possible that if the program was longer, an autonomy supportive climate might have affected the intent to run in the future.

Unlike future intent to run, it appeared that the autonomy supportive motivational climate did affect run performance. Most notable is the significant difference found between the September and December laps run in the treatment group but not in the control group. This finding supports our secondary hypothesis that the participants exposed to the autonomy supportive motivational climate would experience overall increased individual run performances. Interpreted from an SDT standpoint, autonomy supportive conditions are conducive towards an internal locus of causality, that is, the belief that one can exert influence or control over the outcome of events [20]. Hence, the faster running times in the autonomy condition may have been the result of the belief that one could achieve enhanced running times through personal effort and enhanced motivation to run farther during the timed runs. Or perhaps, there were more participants in the treatment group who had a lot more room for physical improvement than the control participants.

Results also revealed a gender difference on the Cooper 12-minute run test at both schools. From the September run results, overall, the males ran 14.52 laps whereas the females ran 11.67. At the time of the final run in December, the males ran 15.27 laps whereas the females ran 12.76 laps. This finding supports previous research that males are not only more physically active than females, but also demonstrate increased levels of physical fitness [30–32]. Previous research has also found that when implementing various types of running programs, run times (or in the current research—number of laps run) improve from the beginning of the program to the end regardless of the environment [4, 12, 33]. Participants improve in cardiovascular fitness because they practice running.

Although the findings from this study provided support for the benefits of an autonomy supportive environment with regards to improved running performance, this study is not without limitations. The measure of future intent was a self-report questionnaire taken immediately upon completion of the running programs. Future studies should consider using postintervention followups of 6 months to a year to more specifically determine if participants did indeed continue to run. In addition, although the length of time running was increased throughout the program, no measure of intensity was utilized. It would have been interesting to have had the students wear heart rate monitors and/or accelerometers to obtain a better measure of daily running intensity throughout the programs.

The primary implication for school physical activity professionals is that both running programs resulted in increased future intent to run and improved run performance. This provides support for the implementation of established school running programs such as “Girls on the Run” and “ING Run for Something Better” or for any general before/during/after-school running program. Given the lack of financial resources available to many schools, particularly those located in lower income areas, running programs offer both a cost- and time-efficient alternative to more costly sport-based activity programs.

## Figures and Tables

**Table 1:** Overall pre- and posttest future intent score means.

Participants	Pretest *M* ± SD	Posttest *M* ± SD
Treatment		
Males (*n* = 30)	3.72 ± 1.19	3.98 ± 0.78
Females (*n* = 39)	3.62 ± 0.76	3.80 ± 1.05
Total (*n* = 69)	3.66 ± 0.94	3.87 ± 0.95
Control		
Males (*n* = 72)	3.81 ± 0.91	3.89 ± 0.90
Females (*n* = 59)	3.61 ± 1.13	3.75 ± 1.30
Total (*n* = 129)	3.73 ± 1.00	3.83 ± 0.95
Treatment + control total	3.70 ± 0.98	3.84 ± 0.95*

This table shows the overall future intent means for both genders at both schools for both the pretest and posttest. *Significant difference pre-post test *P* = .026.

**Table 2:** Monthly lap run means.

Run	Gender	Group	*M *
September	Male	Treatment	13.593
		Control	14.847
	Female	Treatment	10.098
		Control	12.856

October	Male	Treatment	12.581
		Control	15.646
	Female	Treatment	10.939
		Control	13.417

November	Male	Treatment	13.875
		Control	14.839
	Female	Treatment	11.146
		Control	13.208

December	Male	Treatment	14.796
		Control	15.433
	Female	Treatment	12.537
		Control	12.935

This table shows the monthly lap run means for each school by gender.

**Table 3:** Paired monthly runs.

School	Pair	*M *	SD	95% confidence level		df	Sig. (2-tailed)
				Lower	Upper		
Treatment	Sept-Oct	−0.089	3.091	−0.832	0.653	68	0.810
	Sept–Nov	−0.719	2.948	−1.423	−0.011	68	0.047
	Sept–Dec	−1.877	5.252	−3.139	−0.615	68	0.004^#^
	Oct-Nov	−0.629	3.565	−1.486	0.227	68	0.147
	Oct–Dec	−1.787	4.611	−2.895	−0.679	68	0.002^#^
	Nov-Dec	−1.158	4.140	−2.152	−0.163	68	0.023

Control	Sept-Oct	−0.699	1.864	−1.024	−0.374	128	0.000^#^
	Sept–Nov	−0.143	2.543	−0.586	0.300	128	0.525
	Sept–Dec	−0.374	2.236	−0.764	0.016	128	0.060
	Oct-Nov	0.556	2.358	0.145	0.967	128	0.008
	Oct–Dec	0.325	2.058	−0.033	0.684	128	0.075
	Nov-Dec	−0.231	2.475	−0.662	0.199	128	0.291

^#^Alpha-adjusted significant at alpha = .004.
